# Supplementary material for: Detection of Enterobius vermicularis in archived formalin-fixed paraffin-embedded (FFPE) appendectomy blocks: It’s potential to compare genetic variations based on mitochondrial DNA (cox1) gene
Source: PLoS One. 2023 Feb 9;18(2):e0281622. doi: 10.1371/journal.pone.0281622 (PMC9910638; doi:10.1371/journal.pone.0281622)
Supplement: S1 Table — (PDF) [file pone.0281622.s001.pdf]

S1 Table. The result of the identity and coverage of the sequences obtained from the *cox*1 gene of this study with the isolates of the World GenBank.

Block. NO	Place of Isolation City/Province	ACC.NO	Place of Isolation	ACC.NO	Species	Identity %	Query Coverage %
1	Hamedan	MZ361991	Iran (Khorramabad)	MH802605	*Enterobius vermicularis*	100	100
2	Hamedan	MZ361992	Iran (Shiraz)	MH802604	*Enterobius vermicularis*	100	100
3	Hamedan	MZ361993	Iran (Shiraz)	MH802610	*Enterobius vermicularis*	98.94	100
4	Hamedan	MZ361994	Iran (Shiraz)	MH802609	*Enterobius vermicularis*	98.94	100
5	Hamedan	MZ361995	Iran (Shiraz)	MH802608	*Enterobius vermicularis*	98.68	100
7	Hamedan	MZ361996	Iran (Tabriz)	KJ780777	*Enterobius vermicularis*	98.37	97
9	Hamedan	MZ361997	Japan	AP017684	*Enterobius vermicularis*	98.41	99
14	Hamedan	MZ361998	Czech	FR687965	*Enterobius vermicularis*	98.15	99
15	Hamedan	MZ361999	Thailand	MH208474	*Enterobius vermicularis*	98.32	94
13	Tehran	MZ360956	Iran (Shiraz)	MH802607	*Enterobius vermicularis*	98.94	100
16	Tehran	MZ360957	Iran (Shiraz)	MH802600	*Enterobius vermicularis*	98.94	100
17	Tehran	MZ360958	Iran (Shiraz)	MH802611	*Enterobius vermicularis*	99	100
100	Mazandaran	MZ362434	Iran (Shiraz)	MH802605	*Enterobius vermicularis*	86	99.47
