# Supplementary material for: Detection of Enterobius vermicularis in archived formalin-fixed paraffin-embedded (FFPE) appendectomy blocks: It’s potential to compare genetic variations based on mitochondrial DNA (cox1) gene
Source: PLoS One. 2023 Feb 9;18(2):e0281622. doi: 10.1371/journal.pone.0281622 (PMC9910638; doi:10.1371/journal.pone.0281622)
Supplement: S2 Table — (PDF) [file pone.0281622.s002.pdf]

S2 Table. Sequences used in phylogeny tree, polymorphism, and haplotype network analysis according to haplotype cluster, host, and country.

Haplotype cluster	GenBank Accession numbers	Host	Country
B	AB626863	Homo sapience	Sudan
B	HQ395271	Homo sapience	Greece
B	HQ317432	Homo sapience	Greece
B	HQ395270	Homo sapience	Greece
B	HQ317429	Homo sapience	Greece
B	HQ317439	Homo sapience	Greece
B	HQ317440	Homo sapience	Greece
B	HQ317434	Homo sapience	Greece
B	HQ317437	Homo sapience	Greece
B	HQ317438	Homo sapience	Greece
B	HQ317435	Homo sapience	Greece
B	AB221468	Chimpanzees	Japan
B	AB221469	Chimpanzees	Japan
B	AB221467	Chimpanzees	Japan
B	KX527601	Homo sapience	Poland
B	KX527600	Homo sapience	Poland
B	KX527602	Homo sapience	Poland
B	JQ411505	Homo sapience	Germany
B	JQ411509	Homo sapience	Germany
B	JQ411507	Homo sapience	Germany
B	AB626871	Homo sapience	Czech Republic
B	FR687965	Homo sapience	Czech Republic
B	JQ411488	Homo sapience	Denmark
B	JQ411484	Homo sapience	Denmark
B	JQ411497	Homo sapience	Denmark
B	JQ411496	Homo sapience	Denmark
B	JQ411491	Homo sapience	Denmark
B	JQ411489	Homo sapience	Denmark
B	JQ411493	Homo sapience	Denmark
B	JQ411494	Homo sapience	Denmark
B	JQ411504	Homo sapience	Denmark
B	MZ360956	Homo sapience	IRAN
B	MZ360957	Homo sapience	IRAN
B	MZ361998	Homo sapience	IRAN
B	MZ361997	Homo sapience	IRAN
B	MZ361994	Homo sapience	IRAN
B	MZ361993	Homo sapience	IRAN
B	MZ361991	Homo sapience	IRAN
B	MZ362434	Homo sapience	IRAN
B	MZ361999	Homo sapience	IRAN
B	MZ361995	Homo sapience	IRAN
B	MZ361996	Homo sapience	IRAN
B	MZ360958	Homo sapience	IRAN
B	KJ780776	Homo sapience	Iran- Tabriz

B	KJ780777	Homo sapience	Iran- Tabriz
B	MH802604	Homo sapience	Iran- Khorramabad
B	MH802605	Homo sapience	Iran- Khorramabad
B	MH802603	Homo sapience	Iran- Khorramabad
B	MH802596	Homo sapience	Iran- Khorramabad
B	MH802607	Homo sapience	Iran- Shiraz
B	MH802606	Homo sapience	Iran- Shiraz
B	MH802597	Homo sapience	Iran- Shiraz
B	MH802610	Homo sapience	Iran- Shiraz
B	MH802608	Homo sapience	Iran- Shiraz
B	MH802609	Homo sapience	Iran- Shiraz
B	MH208464	Homo sapience	Thailand
B	MH208474	Homo sapience	Thailand
B	MH208480	Homo sapience	Thailand
B	MH208479	Homo sapience	Thailand
B	MH208465	Homo sapience	Thailand
B	MH208478	Homo sapience	Thailand
B	MH208473	Homo sapience	Thailand
B	MH208477	Homo sapience	Thailand
A	AB221472	Homo sapience	Japan
A	AB221470	Homo sapience	Japan
A	AB221471	Homo sapience	Japan
A	AB221473	Chimpanzees	Japan
A	AB221474	Chimpanzees	Japan
A	EU281143	Homo sapience	Korea
A	MH208467	Homo sapience	Thailand
A	MH208472	Homo sapience	Thailand
A	MH208475	Homo sapience	Thailand
A	MH208466	Homo sapience	Thailand
A	MH208476	Homo sapience	Thailand
A	MH208470	Homo sapience	Thailand
A	MH208469	Homo sapience	Thailand
C	AB221463	Chimpanzees	Japan
C	AB221466	Chimpanzees	Japan
C	AB221464	Chimpanzees	Japan
C	AB221465	Chimpanzees	Japan
C	AB221461	Chimpanzees	Japan
C	AB221462	Chimpanzees	Japan
C	AB221459	Chimpanzees	Japan
C	AB221458	Chimpanzees	Japan
C	AB221457	Chimpanzees	Japan
C	AB221460	Chimpanzees	Japan
