# Supplementary material for: Detection of Enterobius vermicularis in archived formalin-fixed paraffin-embedded (FFPE) appendectomy blocks: It’s potential to compare genetic variations based on mitochondrial DNA (cox1) gene
Source: PLoS One. 2023 Feb 9;18(2):e0281622. doi: 10.1371/journal.pone.0281622 (PMC9910638; doi:10.1371/journal.pone.0281622)
Supplement: S1 Fig — The presence of the asterisk indicates similarity, and its absence indicates the difference in the sequences in that position. (PDF) [file pone.0281622.s003.pdf]

```
MZ360958      TTTGGTCATCCTGAGGTTTATATTCTTATTTTGCCTGCTTTTGGGATTGTTAGTCATAGA   60
MZ362434      TTTGTGCATCCTGAGGTTTATATTCTTATTTTACCTGCTTTTGGGATTGTTAGTCATAGG   60
MZ361995      TTTGGTCATCCTGAGGTTTATATTCTTATTTTACCTGCTTTTGGGATTGTTAGTCATAGG   60
MZ361998      TTTGGTCATCCTGAGGTTTATATTCTTATTTTACCTGCTTTTGGGATTGTTAGTCATAGG   60
MZ361997      TTTGGTCATCCTGAGGTTTATATTCTTATTTTACCTGCTTTTGGGATTGTTAGTCATAGG   60
MZ361994      TTTGGTCATCCTGAGGTTTATATTCTTATTTTACCTGCTTTTGGGATTGTTAGTCATAGG   60
MZ361993      TTTGGTCATCCTGAGGTTTATATTCTTATTTTACCTGCTTTTGGGATTGTTAGTCATAGG   60
MZ361991      TTTGGTCATCCTGAGGTTTATATTCTTATTTTACCTGCTTTTGGGATTGTTAGTCATAGG   60
MZ361996      TTTGGTCATCCTGAGGTTTATATTCTTATTTTACCTGCTTTTGGGATTGTTAGTCATAGG   60
MZ361999      TTTGGTCATCCTGAGGTTTATATTCTTATTTTACCTGCTTTTGGGATTGTTAGTCATAGG   60
MZ360956      TTTGGTCATCCTGAGGTTTATATTCTTATTTTACCTGCTTTTGGGATTGTTAGTCATAGG   60
MZ360957      TTTGGTCATCCTGAGGTTTATATTCTTATTTTACCTGCTTTTGGGATTGTTAGTCATAGG   60
              ****  **************************.**************************.

MZ360958      ATTTTGTGTTTAACTGGTAAAAAGGAGGTGTTTGGTCATTTGGGTATGATTTATGCTATT   120
MZ362434      ATTCTGTGTTTAACTGGTAAAAAGGAGGTGTTTGGTCATTTGGGTATGATTTATGCTATT   120
MZ361995      ATTCTGTGTTTAACTGGTAAAAAGGAGGTGTTTGGTCATTTGGGTATGATTTGTGCTATT   120
MZ361998      ATTCTGTGTTTAACTGGTAAAAAGGAGGTGTTTGGTCATTTGGGTATGATTTATGCTATT   120
MZ361997      ATTCTGTGTTTAACTGGTAAAAAGGAGGTGTTTGGTCATTTGGGTATGATTTATGCTATT   120
MZ361994      ATTCTGTGTTTAACTGGTAAAAAGGAGGTGTTTGGTCATTTGGGTATGATTTATGCTATT   120
MZ361993      ATTCTGTGTTTAACTGGTAAAAAGGAGGTGTTTGGTCATTTGGGTATGATTTATGCTATT   120
MZ361991      ATTCTGTGTTTAACTGGTAAAAAGGAGGTGTTTGGTCATTTGGGTATGATTTATGCTATT   120
MZ361996      ATTCTGTGTTTAACTGGTAAAAAGGAGGTGTTTGGTCATTTGGGTATGATTTATACTATT   120
MZ361999      ATTCTGTGTCTAACTGGTAAAAAGGAGGTGTTTGGTCATTTGGGTATGATTTATGCTATT   120
MZ360956      ATTCTGTGTTTAACTGGTAAAAAGGAGGTGTTTGGTCATTTGGGTATGATTTATGCTATT   120
MZ360957      ATTCTGTGTTTAACTGGTAAAAAGGAGGTGTTTGGTCATTTGGGTATGATTTATGCTATT   120
              *** ***** ******************************************.*.*****

MZ360958      ATTTCTATTGGTTTAATTGGTAGGGTAGTATGGGGTCATCATATGTTTACTATTGGTTTT   180
MZ362434      ATTTCTATTGGTTTAATTGGTAGGGTAGTATGGGGTCATCATATGTTTACTATTGGTTTT   180
MZ361995      ATTTCTATTGGTTTAATTGGTAGGGTAGTATGGGGTCATCATATGTTTACTATTGGTTTT   180
MZ361998      ATTTCTATTGGTTTAATTGGTAGGGTAGTATGGGGTCATCATATGTTTACTATTGGTTTT   180
MZ361997      ATTTCTATTGGTTTAATTGGTAGGGTAGTATGGGGTCATCATATGTTTACTATTGGTTTT   180
MZ361994      ATTTCTATTGGTTTAATTGGTAGGGTAGTATGGGGTCATCATATGTTTACTATTGGTTTT   180
MZ361993      ATTTCTATTGGTTTAATTGGTAGGGTAGTATGGGGTCATCATATGTTTACTATTGGTTTT   180
MZ361991      ATTTCTATTGGTTTAATTGGTAGGGTAGTATGGGGTCATCATATGTTTACTATTGGTTTT   180
MZ361996      ACTTCTATTGGTTTAATTGGTAGGGTAGTATGGGGTCATCATATGTTTACTATTGGTTTT   180
MZ361999      ATTTCTATTGGTTTAATTGGTAGGGTAGTATGGGGTCATCATATGTTTACTATTGGTTTT   180
MZ360956      ATTTCTATTGGTTTAATTGGTAGGGTAGTATGGGGTCATCATATGTTTACTATTGGTTTT   180
MZ360957      ATTTCTATTGGTTTAATTGGTAGGGTAGTATGGGGTCATCATATGTTTACTATTGGTTTT   180
              * **********************************************************

MZ360958      GATATAAGAACACGTTTGTATTTTATGGTTGCTACTATAATTATTGCTGTGCCAACTGGG   240
MZ362434      GATATAAGAACACGTTTGTATTTTATGGTTGCTACTATAATTATTGCTGTGCCAACTGGG   240
MZ361995      GATATAAGAACACGTTTGTATTTTATGGTTGCTACTATAATTATTGCTGTGCCAACTGGG   240
MZ361998      GATATAAGAACACGTTTGTATTTTATGGTTGCTACTATAATTATTGCTGTGCCAACTGGG   240
MZ361997      GATATAAGAACACGTTTGTATTTTATGGTTGCTACTATAATTATTGCTGTGCCAACTGGG   240
MZ361994      GATATAAGAACACGTTTGTATTTTATGGTTGCTACTATAATTATTGCTGTGCCAACTGGG   240
MZ361993      GATATAAGAACACGTTTGTATTTTATGGTTGCTACTATAATTATTGCTGTGCCAACTGGG   240
MZ361991      GATATAAGAACACGTTTGTATTTTATGGTTGCTACTATAATTATTGCTGTGCCAACTGGG   240
MZ361996      GATATAAGAACACGTTTGTATTTTATGGTTGCTACTATAATTATTGCTGTGCCAACTGGG   240
MZ361999      GATATAAGAACACGTTTGTATTTTATGGTTGCTACTATAATTATTGCTGTGCCAACTGGG   240
MZ360956      GATATAAGAACACGTTTGTATTTTATGGTTGCTACTATAATTATTGCTGTGCCAACTGGG   240
MZ360957      GATATAAGAACACGTTTGTATTTTATGGTTGCTACTATAATTATTGCTGTGCCAACTGGG   240
```

```
            ************************************************************

MZ360958    GTAAAGGTTTTTAGTTGGTTGTTAACTTTGATAGGGGGACGTTTAGTTGTGCAGCCTTTA   300
MZ362434    GTAAAGGTTTTTAGTTGGTTGTTGACTTTGATAGGGGGACGTTTAGTTGTGCAGCCTTTA   300
MZ361995    GTAAAGGTTTTTAGTTGGTTGTTGACTTTGATAGGGGGACGTTTAGTTGTGCAGCCTTTA   300
MZ361998    GTAAAGGTTTTTAGTTGGTTGTTGACTTTGATAGGGGGACGTTTAGTTGTGCAGCCTTTA   300
MZ361997    GTAAAGGTTTTTAGTTGGTTGTTGACTTTGATAGGGGGACGTTTAGTTGTGCAGCCTTTA   300
MZ361994    GTAAAGGTTTTTAGTTGGTTGTTGACTTTGATAGGGGGACGTTTAGTTGTGCAGCCTTTA   300
MZ361993    GTAAAGGTTTTTAGTTGGTTGTTGACTTTGATAGGGGGACGTTTAGTTGTGCAGCCTTTA   300
MZ361991    GTAAAGGTTTTTAGTTGGTTGTTGACTTTGATAGGGGGACGTTTAGTTGTGCAGCCTTTA   300
MZ361996    GTAAAGGTTTTTAGTTGGTTGTTGACTTTGGTAGGGGGACGTTTAGTTGTGCAGCCTTTA   300
MZ361999    GTAAAGGTTTTTAGTTGGTTGTTGACTTTGATAGGGGGACGTTTAGTTGTGCAGCCTTTA   300
MZ360956    GTAAAGGTTTTTAGTTGGTTGTTGACTTTGATAGGGGGACGTTTAGTTGTGCAGCCTTTA   300
MZ360957    GTAAAGGTTTTTAGTTGGTTGTTGACTTTGATAGGGGGACGTTTAGTTGTGCAGCCTTTA   300
            ***********************.******.*****************************

MZ360958    TTGTTTTGGGTTTTGGGGTTTATTTTTATGTTTACTACGGGGGGTTTAACAGGTATTATG   360
MZ362434    TTGTTTTGGGTTTTGGGGTTTATTTTTATGTTTACTACGGGGGGTTTAACAGGTATTATG   360
MZ361995    TTGTTTTGGGTTTTGGGGTTTATTTTTATGTTTACTACGGGGGGTTTAACAGGTATTATG   360
MZ361998    TTGTTTTGGGTTTTGGGGTTTATTTTTATGTTTACTACGGGGGGTTTAACAGGTATTATG   360
MZ361997    TTGTTTTGGGTTTTGGGGTTTATTTTTATGTTTACTACGGGGGGTTTAACAGGTATTATG   360
MZ361994    TTGTTTTGGGTTTTGGGGTTTATTTTTATGTTTACTACGGGGGGTTTAACAGGTATTATG   360
MZ361993    TTGTTTTGGGTTTTGGGGTTTATTTTTATGTTTACTACGGGGGGTTTAACAGGTATTATG   360
MZ361991    TTGTTTTGGGTTTTGGGGTTTATTTTTATGTTTACTACGGGGGGTTTAACAGGTATTATG   360
MZ361996    TTGTTTTGGGTTTTGGGGTTTATTTTTATGTTTACTACGGGGGGTTTAACAGGTATTATG   360
MZ361999    TTGTTTTGGGTTTTGGGGTTTATTTTTATGTTTACTACGGGGGGTTTAACAGGTATTATG   360
MZ360956    TTGTTTTGGGTTTTGGGGTTTATTTTTATGTTTACTACGGGGGGTTTAACAGGTATTATG   360
MZ360957    TTGTTTTGGGTTTTGGGGTTTATTTTTATGTTTACTACGGGGGGTTTAACAGGTATTATG   360
            ************************************************************

MZ360958    TTGGCTAATCCTATTTTG                                             378
MZ362434    TTGGCTAATCCTATTTTA                                             378
MZ361995    TTGGCTAATCCTATTTTG                                             378
MZ361998    TTGGCTAATCCTATTTTG                                             378
MZ361997    TTGGCTAATCCTATTTTG                                             378
MZ361994    TTGGCTAATCCTATTTTG                                             378
MZ361993    TTGGCTAATCCTATTTTG                                             378
MZ361991    TTGGCTAATCCTATTTTG                                             378
MZ361996    TTGGCTAATCCTATTTTG                                             378
MZ361999    TTGGCTAATCCTATTTTG                                             378
MZ360956    TTGGCTAATCCTATTTTG                                             378
MZ360957    TTGGCTAATCCTATTTTG                                             378
            *****************.
```

S1 Fig. Alignment of 378 bp of the nucleotide sequence of the *cox*1 gene fragment isolated from the paraffin blocks and the adult worm obtained from this study. The presence of the asterisk indicates similarity, and its absence indicates the difference in the sequences in that position.
