# Supplementary figures and images for: Detection of Enterobius vermicularis in archived formalin-fixed paraffin-embedded (FFPE) appendectomy blocks: It’s potential to compare genetic variations based on mitochondrial DNA (cox1) gene
Source: PLoS One. 2023 Feb 9;18(2):e0281622. doi: 10.1371/journal.pone.0281622 (PMC9910638; doi:10.1371/journal.pone.0281622)

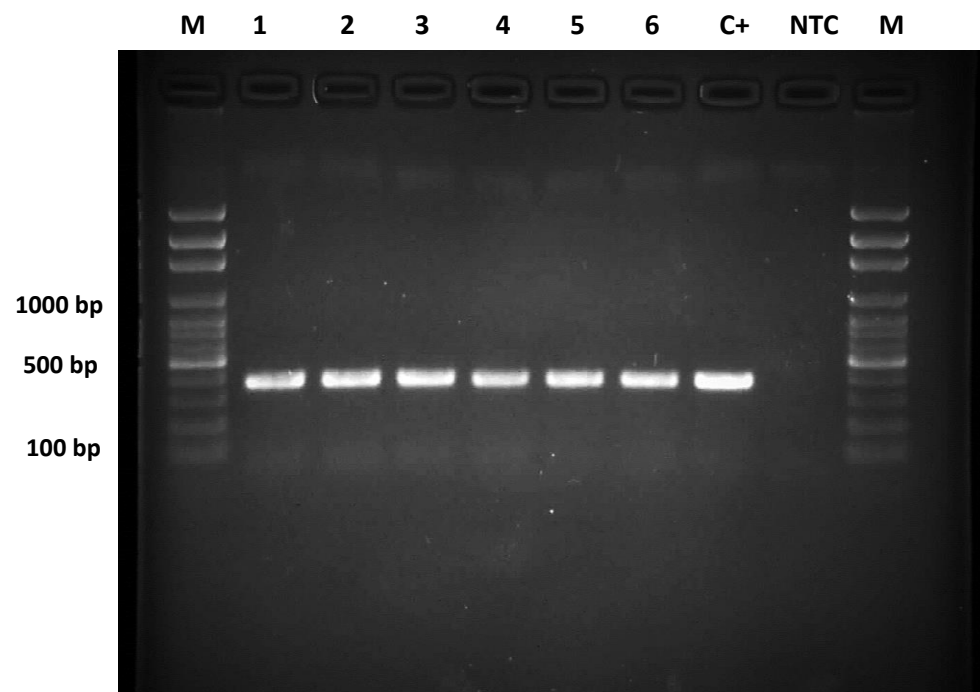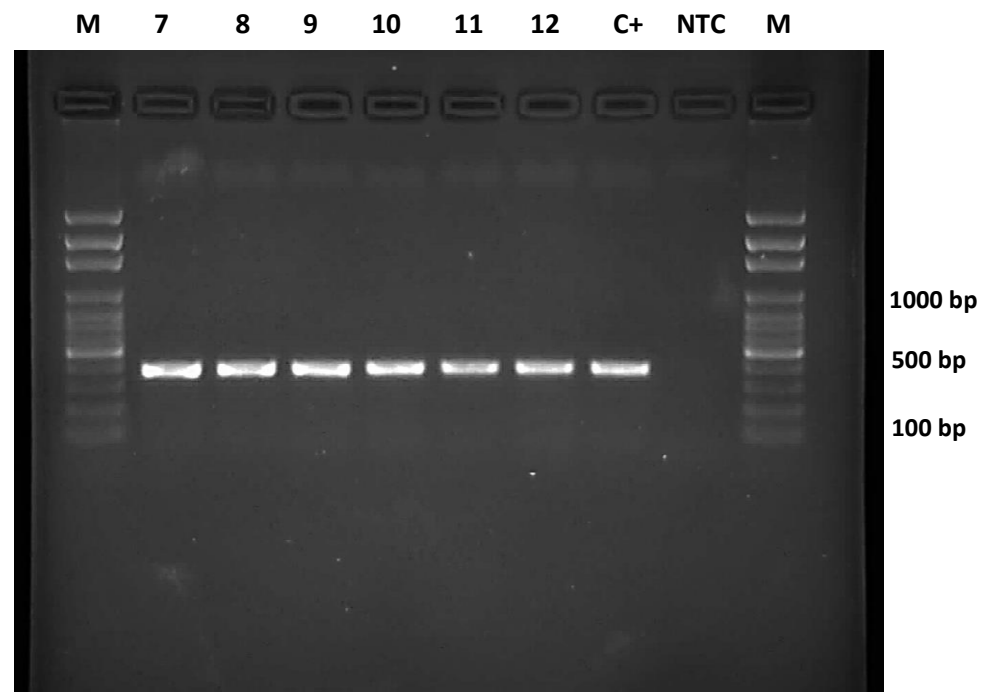

Supplement: S1 Raw images — (PDF) [file pone.0281622.s004.pdf]
