# Supplementary material for: Detection of Enterobius vermicularis in archived formalin-fixed paraffin-embedded (FFPE) appendectomy blocks: It’s potential to compare genetic variations based on mitochondrial DNA (cox1) gene
Source: PLoS One. 2023 Feb 9;18(2):e0281622. doi: 10.1371/journal.pone.0281622 (PMC9910638; doi:10.1371/journal.pone.0281622)
Supplement: Attachment — Submitted filename: Enterobius 18th September final (1).docx [file pone.0281622.s005.docx]

**Detection of *Enterobius vermicularis* in archived formalin-fixed paraffin-embedded (FFPE) appendectomy blocks: It's potential to compare genetic variations based on mitochondrial DNA (*cox*1) gene**

**Maryam Haghshenas^1^, Mona Koosha^2^, Alireza Latifi^1^, Elham Kazemirad^1^*, Arash Dehghan^3^, Bahram Nikmanesh^4^, Gholamreza Mowlavi^1,5^***

^1^ Department of Medical Parasitology & Mycology, School of Public Health & Institute of Public Health Research, Tehran University of Medical Sciences

^2^ Department of Medical Entomology and Vector Control, School of Public Health, Tehran University of Medical Sciences

^3^ Department of Pathology, School of Medicine, Hamedan University of Medical Sciences, Hamedan, Iran

^4^ Department of Medical Laboratory Sciences, School of Allied Medical Sciences, Tehran University of Medical Sciences, Tehran, Iran.

^5^ Center for Research of Endemic Parasites of Iran (CREPI), Tehran University of Medical Sciences, Tehran, Iran

**Correspondence to:**

**Gholamreza Mowlavi,** [**molavig@yahoo.com**](mailto:molavig@yahoo.com)

**Elham Kazemirad,** [**Kazemirad@tums.ac.ir**](mailto:Kazemirad@tums.ac.ir)**,** [**ekazemirad@yahoo.com**](mailto:ekazemirad@yahoo.com)

# Abstract

Acute appendicitis represents one of the most common causes of emergency abdominal surgery worldwide. Enterobius vermicularis has been suggested as one of the probable causes of appendicitis. In this study, the morphological characteristics of the remnant pinworms and pathologic changes were explored in old-archived FFPE tissues of appendectomies. Moreover, we provide the first molecular identification, genetic, and haplotype variation of this nematode from the old-archived FFPE tissue section of appendectomy using the mitochondrial cytochrome c oxidase subunit 1 (*cox*1) gene. Seventeen FFPE appendectomy tissue from patients with appendicitis caused by *E. vermicularis* were collected from two different geographical areas of Iran. 14 (82.3%) samples were related to 20-22 years ago, and 3 (17.6%) samples were 12-16 years old. After histopathologic examination, DNA was extracted from these blocks, and the adult worm and *cox*1 gene were amplified using the nested polymerase chain reaction (PCR). Thirteen amplified PCR products (378 bp) were sequenced and followed by Phylogenetic analysis. In the histopathological examination, tissue changes were observed in 13 cases (76.4%) and inflammation just in 4 blocks (23.5%). Of the 17 positive FFEP samples, 12 (70.6%) cases were amplified successfully by nested PCR. Phylogenetic analysis and a median-joining network of 78 available *cox*1 sequences of *E. vermicularis* revealed 59 haplotypes. We identified five haplotypes that fell into type B. All Haplotypes are novel except for two haplotypes, 32 and Hap37, which were identical to a previously isolated *E. vermicularis* from Iran, Greece, and Germany. The ranges of diversity distance and haplotype diversity within the isolates were 0-1.9% and HD:0.643- 0.667, subsequently. Overall, the absence of inflammation or even tissue changes in some sections can drag the minds to the possible non-inflammatory role of *E. vermicularis* in causing appendicitis. Although FFPE material suffers from PCR inhibition, we could successfully use Nested PCR to characterize *E. vermicularis* in old-archived bocks and suggest this method as a complementary pathology technique in diagnosing appendicitis due to *E. vermicularis*. The predominant type was B in the Middle East and Europe; however, further studies on a large sample size from different regions could probably confirm the results obtained in the present study.

**Keywords:** *Enterobius vermicularis*, archived Formalin-Fixed Paraffin-Embedded (FFPE) blocks of appendectomies, mitochondrial DNA (*cox*1) gene, genetic diversity, haplotype network

# Introduction

*Enterobius vermicularis* is one of the helminths mentioned as a possible causative agent of appendicitis [1]. It is distributed worldwide and is probably one of the most common helminth infections in humans, with prevalence rates of 30-50 % [2]. The most affected age group was seen among children aged 5-10 years, accounting for more than 30% of all infected cases [3]. Although the *E. vermicoularis* adult females inhabit the cecum of the large intestine, their biological traits in migrating to the perianal skin for laying eggs, mainly at night, can facilitate the retro infection and the autoinfection inhalation route of enterobiasis [3]. Usually, the infection transmission occurs through picking up the eggs by the fingers during the scratching due to perianal pruritus. Meanwhile, the prevalence rate of infestation has been recorded to be high in institutional populations and within family contacts [4]. It has been reported that parasitized children with *E. vermicularis* mostly exhibit a characteristic itch in the perianal area, causing them to carry the eggs by hand [3]. Nevertheless, along with the harmless course of enterobiasis, which has been documented as asymptomatic in almost 40% of infected ones [5], some severe conditions in kidneys and fallopian tubes involvement due to pinworm infection that has led to death for some, should not be neglected [6]. Along with the high-risk prevalent clinical manifestations in childhood that some might be assumed to be associated with the incidence of parasitic infections, acute appendicitis due to enterobiasis has remained controversial in this regard [1, 7]. At the beginning of the 21st century, the incidence of appendicitis was seen to grow in newly industrialized countries. In contrast, in western countries, mortality and morbidities, as well as the imposed economic impact associated with appendicitis, have been reported [8]. Regarding the first description of appendicitis by Fits for the first time more than 100 years ago, reminding of 561,000 performances of appendectomies per year between 1979-1984 in the United States that might have been caused by different etiological agents is worth mentioning [9]. Meanwhile, the influencing factors like low fiber content in the diet, hygiene, ethnic and genetic tendency, socioeconomic status, seasonal variation, temperature, and altitude, along with bacterial, viral, and parasitic infections, have been investigated in appendicitis so far [4]. With an overview of the parasitic worms mentioned as possible etiological agents in appendicitis, the name *E. vermicularis* is the most highlighted, with a more special incidence in children and young adults [10].

In the last decades, limited target genes have been used to explore the genetic diversity, phylogeography, and host specificity of *E. vermicularis*. Both mitochondrial DNA (mtDNA) and ribosomal DNA, such as Internal transcribed spacer 2 (ITS2) regions, were used in phylogenetic analyses, with mitochondrial genes being more appropriate for population genetic studies, whereas ITS remains better for identification [11]. Cytochrome c oxidase subunit 1 (*cox*1) gene is one of the most diverse mitochondrial genes previously used for other nematodes [12, 13]. To date, *cox*1 sequences of *E. vermicularis* have been published in only a few countries. The analysis of the mitochondrial *cox*1 gene sequences in eggs and adult worms from humans and captive chimpanzees (*Pan troglodytes*) in Japan has shown the presence of three different genetic types, designated as type A, B, and C [14]; type A has been identified in both species, whereas types B and C only in chimpanzees. In contrast, based on the sequencing of *cox*1 from *E. vermicularis* eggs on adhesive-tape swabs from humans in Greece, Denmark, Poland, Germany, and Iran, all the isolates were grouped in type B [15-17]. In a recent study in Thailand, types A and B of *E. vermicularis* were isolated from humans by adhesive tape perianal swab technique, suggesting type A might be restricted to eastern Asia [18].

To date, no studies have been conducted on FFPE tissue from appendectomy to explore molecular characterization and genetic variation of *E. vermicularis*. The FFPE tissues are valuable resources preserving the tissues with intact morphological and tissue structures. However, due to the high fragmentation of DNA, successful amplification of DNA is quite challenging and it needs shorter amplification products of 250 to 300 bp in length or higher sensitive techniques such as nested PCR [19, 20]. In the present study, from the etiological point of view, nested PCR was conducted on the *cox*1 gene to confirm the presence of *E. vermicularis* in old-archived FFPE appendicitis tissues. Also, we tried to use a higher amplicon length of up to 378 bp to characterize the possible genomic and haplotype variations among all the deposited *cox*1 sequences in GenBank. Moreover, we explored the morphological characteristics of the remnant pinworms and identified pathological changes in archived appendectomies in FFPE belonging to two different geographical areas in Iran.

# Materials and Methods

## **Sample collection**

This study was approved by the Ethical Committee of the Tehran University of Medical Sciences, Iran. The referred ethics committee waived the need for consent to utilize tissue samples.

We used 17 formalin-fixed paraffin-embedded (FFPE) samples, 14 blocks from Besat Hospital in Hamadan, western Iran, and three blocks from Tehran Medical Center Hospital, Iran, by reviewing the archived files of patients with appendicitis caused by *E. vermicularis* (Table 1). Confirming *E. vermicularis* as found agent in appendectomies was based on observing the eggs or the remaining parts of the worms in the tissue sections. Demographic and clinical features were reflected in the medical records, and gross and histological findings were also regarded.

**Histotechniqucal procedure**

Tissue processing and standard Hematoxylin and eosin staining (H&E) were performed [21]. After identifying a parasitic structure in the stained section, the pathological appearances were determined for each case. An adult form of *E. vermicularis* preserved in 70% alcohol was also used as a standard positive sample; It should be noted that this sample was collected from a patient in Mazandaran province, Iran. Afterward, the samples were cut with a microtome in thickness of 5 to 10 μm and stained with hematoxylin-eosin (H&E) and mounted on a glass slide for microscopic observation. In the end, the morphological and pathological features of the adult worm were checked using light microscope images at different magnifications.

## **DNA extraction**

DNA was extracted from formalin-fixed paraffin-embedded (FFPE) tissue samples, 14 (82.3%) samples related to 20-22 years ago, and 3 (17.6%) samples were 12-16 years old (Table 1). Three serial sections of 8-10-micrometer thickness per sample were taken using a standard microtome (Leica SM2000 R Sliding Microtome, Wetzlar, Germany). The tissue sections from each block were deparaffinized on glass with xylene and washed and rehydrated with descending concentrations of ethanol after incubation at 56 C^o^ for 30 minutes. Tissues were dried at room temperature after washing them in distilled water. Without letting the tissues over-dry, each section was scraped into a 1.5 ml microcentrifuge tube with a sterile scalpel. The tissue samples were digested with tissue lysis buffer and proteinase k (20 mg/ml). The manufacturer's instructions were followed for DNA isolation with the High Pure PCR template preparation kit (Roche, Germany). The final elution volume was 60 µl, and all extracted DNA was stored at -20°C.

## **Nested PCR method**

The targeted DNA regions were amplified for the mitochondrial cytochrome c oxidase 1 (*cox*1) gene by a nested PCR method using the outer primers. CO1 F (5′-TGGTTTTTTGTGCATCCTGAGGTTTA-3′), CO1 R (5′-AGAAAGAACGTAATGAAAATGAGCAAC-3′) [22], and inner primers EVIF (5-TTGGTCATCCTGAGGTTTATATTC-3), EVIR (5-TCCAAAATAGGATTAGCCAACA-3) [23]. The outer and inner amplifications' product sizes were 439 bp and 380 bp, respectively. All cycling conditions were as follows: initial denaturation at 94°C for 6 min, followed by 45 cycles of 94°C for 1 min, 58.2°C for 1 min, and 72°C for 1 min; and a final elongation step at 72°C for 10 min. Two µl of the outer PCR reaction were used as a template for the inner PCR. Conditions applied to the inner PCR were the same as for the outer, with the exception that the annealing temperature was set at 50.1°C and the number of cycles was reduced to 35. Also, we reduced PCR inhibition by increasing the polymerase concentration, dNTP concentration, and PCR elongation time. Positive controls containing DNA extracted from an expelled *E. vermicularis* worm and negative controls containing DNA-free water were included in all runs. Also, DNA from negative control corresponded to appendicitis tissue obtained from surgical specimens of a patient with other etiological agents used.

PCR products were separated with 1.5% agarose gel electrophoresis containing Green Viewer and were photographed using the Vilber Lourmat gel documentation system.

Of the 17 isolates, Successful amplicons were sequenced bidirectionally via Genetic Codon Company, Tehran, Iran, using the inner primers set. (EVIF/R).

## **Analysis of sequence and population genetics**

The quality of crude sequences was improved using the Chromas 2.6.6 program by removing areas with poor quality at both ends of the sequences. The consensus of confident sequences was examined using the NCBI (nucleotide collection) database. Multiple DNA sequences were aligned using the ClustalW program (http://www.ebi.ac.uk/clustalw/) and afterward trimmed. Finally, a 333 bp consensus sequence length was used for phylogenetic analysis. Relationships were analyzed by Maximum Likelihood, Neighbor-Joining, and UPGMA (unweighted pair group method with arithmetic mean) inference methods embedded in MEGA X. The best-fit substitution model for the *cox*1 alignment was the Hasegawa–Kishino–Yano model (HKY+G). The topology of the phylogenetic tree was evaluated using the bootstrap test based on 1000 replications. For phylogenetic analysis, we used 91 sequences, including 75 *cox*1 sequences of *E. vermicularis* available in GenBank, 12 sequences from the current study, as well as those of *Ascaris suum* (accession no. X54253), *Trypanoxyuris atelis* (accession no. AB222177), *Trypanoxyuris microon* (accession no. AB222176) and *Enterobius anthropopitheci* (accession no. AB254450).

The genetic diversity indices, such as haplotype diversity (HD) and nucleotide diversity (π), were calculated by using DnaSP version 6.12.03 [24] in the isolates of *E. vermicularis*. Also, an input file for drawing a median-joining network in PopART software version 1.7 was prepared [25]. Intra- and interspecific genetic differences were calculated with MEGA version X [26, 27] by using the Kimura two-parameter (K2P) distance model [26]. Ninety-one *cox*1 sequences (12 sequences from this study, the remainder from Korea, Japan, Thailand, Iran, Czech Republic, Greece, Germany, Denmark, and Sudan obtained from GenBank) derived from human and chimpanzee hosts were used in the analysis. The sequences reported in this paper were deposited in GenBank.

# Results

# Demographic characteristics of appendectomy sections

Pathological diagnoses of the selected cases were appendicitis due to *E. vermicularis*. Eleven cases (64.7%) occurred in the age group under 20 years, and 6 cases (35.2%) in the patients over 20 years of age. The highest number of appendicitis was related to the age group under 20 years, which can be justified due to the prevalence of this disease at a younger age.

Females with 10 cases (58.8%) out of 17 were seen as the predominant gender of appendicitis due to *E. vermicularis* (Table 1). It is worth mentioning that 14 paraffin-blocked samples (82.3%) were derived from the old archives of 20-22 years ago, 3 (17.6%) samples from 12-16 years old, and the oldest cases belonged to 1998. (Table 1).

**Table 1: Description of appendectomy sections based on age, Pathological, and molecular results.**

| Number of blocks | Code blocks | Relevant hospital, province | Block history | Gender | Age of patient | | Pathological outcome | | Nested PCR *cox*1 | Accession No |
| --- | --- | --- | --- | --- | --- | --- | --- | --- | --- | --- |
|  |  |  |  |  |  |  | **Inflammation** | **Tissue changes** |  |  |
| 1 | **77-648** | **Besat Hospital, Hamedan** | **1998** | **Female** | | **18** | **-** | **+** | **+** | **MZ361991** |
| 2 | **77-649** | **Besat Hospital, Hamedan** | **1998** | **Female** | | **13** | **-** | **-** | **+** | **MZ361992** |
| 3 | **79-475** | **Besat Hospital, Hamedan** | **2000** | **Male** | | **14** | **+** | **+** | **+** | **MZ361993** |
| 4 | **77-846** | **Besat Hospital, Hamedan** | **1998** | **Male** | | **1** | **+** | **+** | **+** | **MZ361994** |
| 5 | **77-143** | **Besat Hospital, Hamedan** | **1998** | **Female** | | **30** | **-** | **+** | **+** | **MZ361995** |
| 6 | **77-667** | **Besat Hospital, Hamedan** | **1998** | **Male** | | **11** | **-** | **-** | **-** | **-** |
| 7 | **79-478** | **Besat Hospital, Hamedan** | **2000** | **Male** | | **6** | **+** | **+** | **+** | **MZ361996** |
| 8 | **79-367** | **Besat Hospital, Hamedan** | **2000** | **Female** | | **20** | **-** | **+** | **-** | **-** |
| 9 | **77-483** | **Besat Hospital, Hamedan** | **1998** | **Female** | | **35** | **-** | **+** | **+** | **MZ361997** |
| 10 | **79-339** | **Besat Hospital, Hamedan** | **2000** | **Male** | | **23** | **+** | **+** | **-** | **-** |
| 11 | **79-535** | **Besat Hospital, Hamedan** | **2000** | **Female** | | **19** | **-** | **+** | **-** | **-** |
| 12 | **77-653** | **Besat Hospital, Hamedan** | **1998** | **Female** | | **25** | **-** | **-** | **-** | **-** |
| 13 | **83-2331** | **Tehran Medical Center** | **2004** | **Male** | | **11** | **-** | **+** | **+** | **MZ360956** |
| 14 | **77-654** | **Besat Hospital, Hamedan** | **1998** | **Male** | | **25** | **-** | **+** | **+** | **MZ361998** |
| 15 | **77-672** | **Besat Hospital, Hamedan** | **1998** | **Female** | | **35** | **-** | **-** | **+** | **MZ361999** |
| 16 | **85-778** | **Tehran Medical Center** | **2006** | **Female** | | **5.5** | **-** | **+** | **+** | **MZ360957** |
| 17 | **87-2013** | **Tehran Medical Center** | **2008** | **Female** | | **11** | **-** | **+** | **+** | **MZ360958** |
| Total |  |  |  | **Female 58.8%**  **Male 41.1%** | |  | **23.5%** | **76.4%** | **70.5%** |  |

## **Pathological findings**

Microscopical detection revealed the inflammation and tissue changes as histopathological findings in some slides. In contrast, in the rest of the sections, the only microscopical output was the presence of the worm remains. Tissue changes were observed in 13 cases (76.5%) and inflammation in 4 blocks (23.5%) (Table 1). In 9 samples (52.94%), no inflammation was observed in the histopathological analysis despite detecting tissue changes. It should be noted that only in 4 samples inflammation and tissue changes were seen together. On the other hand, in 4 initially diagnosed cases with clinically inflamed appendicitis due to *E. vermicularis*, inflammation and prominent tissue changes were not observed in our re-examination. The absence of inflammation or even tissue changes can drag the minds to the possible non-inflammatory role of *E. vermicularis* in causing appendicitis.

The appendix wall can be seen in the histopathological figs 1 (A-C). Lymph follicle hyperplasia with germinal centers in the submucosal layer is apparent. Although the cross-section of *E. vermicularis* worm is seen inside the lumen, there is no evidence of acute appendicitis, which could be controversial pathologically.

## **Diagnostic microanatomy of *Enterobius vermicularis***

Initially, all received slides, and the newly prepared sections were examined microscopically, while the parasitized sections were drawn using Camera Lucida at various magnification. The characteristic morphological appearance of the pinworm has also been exhibited in positive slides (Fig 1). Since the male *E. vermicularis* is rarely seen in patients' samples, it seems that the primary morphological identity of the worm sections should have more focused-on females. According to the cross-section diameter, this photo shows the cross-section of the female *E. vermicularis,* which is about 0.3 to 0.5 mm. The most apparent diagnostic sign in the cross-section of *E. vermicularis* worms is the Cephalic alae which can be seen bilaterally and symmetrically in the sections (Fig 1). Another crucial diagnostic point in the cross-sections of *E. vermicularis* worms is the somatic muscle of the platymyarian and meromyarian types, which are also well visible in the section. In the meromyarian type, the somatic muscles are between two to five in each quadrant in cross-section. Also, platymyarian refers to the compactness of the contractile portion of the somatic muscles. The combination of these morphological signs confirms the diagnosis of *Enterobius vermicularis* worms.

**Fig1.** **Microscopic images of the cross-section of *E. vermicularis* in the appendix lumen in different magnifications (A, B, and C).** Arrows illustrate the cephalic alae located bilateral and symmetrically in the sections. Images were drawn with Camera lucida, *Enterobius vermicularis* cross-section in tissue sections with emphasis on cephalic alae (D, E, and F)

## **Molecular analysis**

### **Nested PCR results**

DNA from FFPT sections was extracted; however, due to the old storage of blocks and possible DNA fragmentation, and little residue of the parasite in the remaining tissue, in the first PCR with COI primers, the results were negative, so the inner primer EVF/R used for successful amplification.

Of the 17 positive paraffin block samples examined, 12 (70.6%) cases produced a band of 378 bp after nested PCR, whereas 5 (29.4%) samples did not have the visible product after reamplification (Fig 2). The oldest cases with positive nested PCR were from 1998.

A total of 12 amplicons of nested PCR related to FFPT tissue were subjected to sequencing. Also, the adult worm of *E. vermicularis* preserved in alcohol 70% successfully was amplified in the first run of PCR with COI primers (439 bp) and sequenced.

**Fig 2.** **Electrophoresis of *cox*1 gene (378 bp) amplified fragments using Nested PCR in paraffin tissue samples of patients with appendicitis due to *E. vermicularis* in Tehran and Hamedan provinces**; M:100 bp DNA Marker (Smobio Technology Inc., Taiwan); lane1: negative control; lanes 2-5: samples and lane 6: Positive control (the adult worm).

### **Genetic variation in the *cox*1 gene of *Enterobius vermicularis* from the appendectomy**

Twelve sequences were deposited in GenBank with accession nos. MZ361991-9, MZ360956-8 (Table 1, S1 Table). In addition, the sequence of a standard sample, adult *E. vermicularis,* preserved in 70% alcohol isolated from a patient in Mazandaran Province, Iran, was also deposited in GenBank with accession no. MZ362434. It should be mentioned that the length of one sequence of Hamedan block after several attempts is very short (277 bp) and did not consider for the genetic variation and phylogenetic analysis.

Twelve sequences (11 from blocks and one from the adult worm), each with 378 bp in the *cox*1 gene, were analyzed for genetic variation (S1 Fig). All the sequence has the highest homology with *Enterobius vermicularis*. Median-Joining haplotype networks analysis demonstrated 12 haplotype sites (3.2%), leading to the characterization of 6 haplotypes among the sequences. Of 12 sequences, seven sequences, including five from Hamedan, and two from Tehran, represent a single haplotype, whereas four sequences of MZ361999 (Hamedan), MZ361996 (Hamedan), MZ361995 (Hamedan), MZ361958 (Tehran), and MZ362434 (Mazandaran) each represent a single haplotype (Fig 3). Using DNAsp, when two provinces were compared, three sequences from Tehran and eight sequences from Hamedan were derived from 2 and 4 haplotypes, respectively, in which one haplotype is common (Table 2). Haplotype diversity of each province, ranging from HD:0.643 to HD: 0.667. In contrast, nucleotide diversity was relatively low for each province, ranging between π:0.00332 for Hamedan patients and π:0.00707 for Tehran patients. Additionally, the mutation rate in polymorphic sites was high in Tehran cases (Θw: 00707) compared to Hamedan cases (0.00512) (Table 2). Of note, all discussed diversity indices in Tehran province were higher than in Hamadan, despite the lower number of cases.

**Fig 3.** **Median-Joining haplotype networks analysis of *cox*1 gene (378 bp) based on 12 isolates of *E. vermicularis* constructed using PopArt 1.7**. Six haplotypes were identified within the isolates in the current study. The details of the sequence(s) belonging to each haplotype are noted. Each circle represents a unique haplotype, n represents the number of sequences in each haplotype, and the hatch marks along the network branches indicate the number of mutations.

**Table 2. Details of statistics genetic diversity for 11 sequences of *E. vermicularis* in Iran.**

| **Provinces** | **Length**  **(bp)** | **Sample size (n)** | **polymorphic sites** | **Number of Haplotypes** | **Haplotype diversity**  **(HD ± SD)** | **Nucleotide diversity**  **(π ± SD)** | **Theta-W**  **(Θw±SD)** | **Nucleotide composition** | |
| --- | --- | --- | --- | --- | --- | --- | --- | --- | --- |
| Hamedan | 377 | 8 | 5 | 4 | 0.643±0.184 | 0.00332±0.00144 | 0.00512±0.00301 | %T | 45.2 |
|  |  |  |  |  |  |  |  | %C | 9.3 |
|  |  |  |  |  |  |  |  | %A | 19.9 |
|  |  |  |  |  |  |  |  | %G | 25.6 |
| Tehran | 377 | 3 | 4 | 2 | 0.667±0.314 | 0.00707±0.00333 | 0.00707±0.00509 | %T | 45.2 |
|  |  |  |  |  |  |  |  | %C | 9.3 |
|  |  |  |  |  |  |  |  | %A | 19.9 |
|  |  |  |  |  |  |  |  | %G | 25.6 |
| Total | 377 | 11 | 12 | ^a^6 | 0.682±0.148 | 0.00531±0.00183 | 0.01054±0.00491 |  |  |

^a^One haplotype is common in Hamadan and Tehran provinces.

^b^Due to the small number of sequences, it was impossible to estimate all indices for Mazandaran cases (access no. MZ362434), so it did not consider for the estimation.

### **Investigating population structure and genetic diversity among isolates of *E. vermicularis***

The genetic distance among the studied samples varies from zero to 0.019 (1.9%), so the highest genetic distance (0.019) was obtained between Tehran samples (MZ360958) and Hamadan samples (MZ361996) as well as between Mazandaran samples (MZ362434) and Tehran samples (MZ360958) (Table 3). The average evolutionary divergence over sequence was estimated according to the Kimura 2-parameter model. Tehran (0.0) and Hamedan (0.01) samples showed high and low evolutionary divergence within the isolates. Likewise, the evolutionary divergence over sequence between Hamedan, Tehran, and Mazandaran isolates based on the Kimura 2-parameter model was also estimated. The highest divergence distance was observed between Mazandaran and Tehran (0.01161) samples and the lowest between Tehran and Hamedan (0.00525) samples (Data not shown).

**Table 3. Pairwise genetic distance matrix of *cox*1 sequences between 12 isolated *Enterobius vermicularis***. Evolutionary analysis was conducted in MEGAX by using the Kimura 2-parameter model. The number of base substitutions per site from between sequences is shown.

| **12** | **11** | **10** | **9** | **8** | **7** | **6** | **5** | **4** | **3** | **2** | **1** |  |  |
| --- | --- | --- | --- | --- | --- | --- | --- | --- | --- | --- | --- | --- | --- |
|  |  |  |  |  |  |  |  |  |  |  |  | **MZ361991** | **1** |
|  |  |  |  |  |  |  |  |  |  |  | 0.000 | **MZ361993** | **2** |
|  |  |  |  |  |  |  |  |  |  | 0.000 | 0.000 | **MZ361994** | **3** |
|  |  |  |  |  |  |  |  |  | 0.003 | 0.003 | 0.003 | **MZ361995** | **4** |
|  |  |  |  |  |  |  |  | 0.011 | 0.008 | 0.008 | 0.008 | **MZ361996** | **5** |
|  |  |  |  |  |  |  | 0.008 | 0.003 | 0.000 | 0.000 | 0.000 | **MZ361997** | **6** |
|  |  |  |  |  |  | 0.000 | 0.008 | 0.003 | 0.000 | 0.000 | 0.000 | **MZ361998** | **7** |
|  |  |  |  |  | 0.003 | 0.003 | 0.011 | 0.005 | 0.003 | 0.003 | 0.003 | **MZ361999** | **8** |
|  |  |  |  | 0.003 | 0.000 | 0.000 | 0.008 | 0.003 | 0.000 | 0.000 | 0.000 | **MZ360956** | **9** |
|  |  |  | 0.000 | 0.003 | 0.000 | 0.000 | 0.008 | 0.003 | 0.000 | 0.000 | 0.000 | **MZ360957** | **10** |
|  |  | 0.011 | 0.011 | 0.013 | 0.011 | 0.011 | 0.019 | 0.013 | 0.011 | 0.011 | 0.011 | **MZ360958** | **11** |
|  | 0.019 | 0.008 | 0.008 | 0.011 | 0.008 | 0.008 | 0.016 | 0.011 | 0.008 | 0.008 | 0.008 | **MZ362434** | **12** |

### **Variation in amino acid sequences**

Variants were found in 5 positions (4%) among the amino acid sequences. As indicated in fig 3, this type of substitution changed the amino acid sequences, particularly in the Hamadan isolate with accession nos. MZ361996 and MZ361995 were novels. All the substitutions were transitions (Fig 4).

There were five variable sites in 126 amino acid sequences, and *E. vermicularis* isolate from Hamedan with accession number MZ361996 with three variable sites is the most variable isolate investigated (Fig 4). In the sequence with accession number 96, two amino acids in positions number 39 and 41 have a new amino acid in type B. In this sequence, the amino acids alanine (A) and isoleucine (I) have been converted to threonine (T), and in both cases, the hydrophobic amino acid has been converted to neutral polar. Amino acid position 38 in the sequence with accession number 95 is also new in type B. In the sequence with accession number 95, the aromatic amino acid tyrosine (Y) has been converted to cysteine (C), which is again neutral polar. These three mutations are new in *E. vermicularis* type B, but the other changes seen in Fig 4 are also detected in the gene bank sequences and are not new. All new substitutions in the sequence of amino acids were non-synonymous substitutions and transitions in the second position of the codon. The highest haplotype diversity in Iranian isolates was mainly due to synonymous substitutions. Amino acid sequences are not different except in two samples where non-synonymous substitutions occurred.

**Fig 4.** **The amino acid alignment of mitochondrial cytochrome c oxidase 1 (*cox*1) gene of Iranian *E. vermicularis* haplotypes with marked changes in the amino acids sequence.** Dots denote identical amino acids to the top sequence.

### **Phylogenetic tree**

Sequences were aligned and trimmed to a length of 333 bp according to previously published *cox*1 sequences [14, 23]. Therefore, the shorter sequences of 333 bp were not considered in molecular studies and phylogenetic trees. Maximum likelihood, neighbor-joining, and UPGMA analysis of mt-DNA *cox*1 data generated the trees with similar topology and equal levels of node support with slightly lower bootstrap values (data not shown). The phylogenetic trees showed that the genus *Enterobius* diverged from the common ancestor of *T. microon* and *T. atelis* at first, and then the ancestors of *E. anthropopitheci* and *E. vermicularis* were separated (Fig 5). Phylogenetic analysis based on 74 *cox*1 sequences (12 from this study and others among the available sequences of GenBank) demonstrated that the *Enterobius vermicularis* divided into three groups like former studies, with predominancy of type B (S2 Table) [14]. As shown in Fig 5, all sequences from this study are clustered within type B. All the isolates from appendectomy blocks and the adult worm were placed in the same clade with reference sequences from Iran except for MZ360958, which was placed in a separate clade with reference sequences from Greece, Germany, and Iran.

Based on the host, Type B in Iran, Greece, Germany, Thailand, Denmark, Sudan, Czech Republic, and Poland was isolated from humans, while in Japan, it was isolated just from chimpanzees. On the other hand, type A in Japan was isolated from humans and chimpanzees, while in South Korea and Thailand, human cases were just reported, and type C was just reported from chimpanzees in Japan (Fig 5, Supplementary Table. S2).

**Fig 5. Phylogenetic relationships were inferred from 333 bp of c*ox1* gene sequences of *Enterobius vermicularis* isolates in this study (black circles) and others retrieved from GenBank.** The tree was constructed based on the maximum likelihood method in MEGAX with *Ascaris suum* as the outgroup. Bootstrap values (>50%) are shown at the nodes based on 1000 replicatesb. The scale bar indicates genetic distance. All the reference sequences of Enterobius vermicularis from GenBank are displayed with their accession number, the host origins, and the original countries.

### **Haplotype network analysis**

In agreement with the phylogenetic tree, all 12 sequences fell into type B and represented five haplotypes (Hap32, Hap37, Hap57, Hap58, Hap59). Three haplotypes, Hap57, Hap58, and Hap59, are novel and were each represented by a single sequence; these haplotypes belonged to the Hamden province, whose accession nos. are MZ361995, MZ361996, and MZ361999, respectively. On the other hand, Hap32 (MZ360958) and Hap37 (8 remaining sequences) were identical to a previously isolated *E. vermicularis* from Iran, Greece, and Germany (Fig 6).

No sequences from Iran fell into types A and C. As shown in Fig 6, 59 haplotypes from different countries are displayed separately as colored bullets. Peripheral haplotypes mainly had single-nucleotide variation (SNV) from central haplotypes. The amount of SNV is plotted as a line bar in Fig 6. Iranian haplotypes with red color were placed next to other haplotypes related to Type B and were separated from haplotypes of Type B and C.

The average evolutionary divergence within and between *E. vermicularis* haplotypes related to the current study and GenBank was estimated according to the Kimura 2-parameter model. It was demonstrated that the evolutionary divergence within the haplotypes of type B is higher than that of types A and C.

Also, the highest divergence distance was observed between Type C and Type A (0.0635), and the lowest was between Type B and Type A (0.0346) haplotypes.

**Fig 6. Median-joining network analysis of haplotype inferred from *cox*1 sequences (333 bp) of *E. vermicularis* by using PopArt 1.7 software.** Eighty-seven sequences were distributed among 59 haplotypes (12 sequences from this study and the rest from other studies in the NCBI database). All the sequences from this study belonged to type B. Each circle represents a haplotype, and its colors represent a country. The size of the circle indicates the relative frequency of sequences belonging to a particular haplotype. Hatch marks along the network branches indicate the number of mutations. B. Distribution and diversity of different haplotypes of *E. vermicularis* based on locations on the world map. Each color represents a different geographical location.

# Discussion

Acute appendicitis is the most common severe abdominal pain requiring emergency surgery [28]. *E. vermicularis* represents one of the controversial etiologic agents for acute appendicitis. A recent meta-analysis reported that *E. vermicularis* had been detected in 4% of pathologic sections of appendicitis [29]. A definitive diagnosis of appendicitis is made through the presence of eggs or larvae worms in pathological examination of surgical specimens.

Meanwhile, paraffin embedding of tissues preserved in formalin could be an appropriate material for molecular identification of parasites, such as *Ascaris* sp [30], *Trypanosoma cruzi* [31], *Leishmania* spp [32], and *Angiostrongylus costaricensis* [33]. Here, for the first time, we used old-archived FFPE tissue of appendectomies for molecular characterization of *E. vermicularis* from two different geographical areas in Iran. Moreover, pathological changes, morphological characteristics, and genetic variation of *E. vermicularis* were precisely studied. In the present study, 11 cases (64.7%) of appendicitis were observed in the age group below 20 years and 6 cases (35.2%) in the group above 20 years (Table 1). The highest number of appendicitis samples was related to the age group under 20 years old, which agrees with the prevalence of this disease at younger ages. Also, with 10 cases (58.8%) out of 17 cases, female was the predominant gender of appendicitis due to *E. vermicularis*. According to a number of studies, the prevalence of *E. vermicularis* in females was higher than in males, which may reflect differences in gender-related contact behavior. Indeed, female housewives have close contact with raw vegetables, making them more susceptible to being infected with parasite eggs [10, 29].

Here, each FFPE sample was examined to determine histopathological changes in which tissue changes were detected in 13 cases (76.4%), while inflammation was observed only in 4 cases (23.5%). It is worth mentioning that in 4 specimens, despite having been diagnosed as appendicitis due to the presence of *E. vermicularis*, no inflammation and tissue changes were detected. This is in favor of the possible role of *E. vermicularis* in non-inflammatory appendicitis. Our pathological study revealed lymph follicle hyperplasia in the submucosal layer; however, there is no sign of classical acute appendicitis, which could be debated pathologically. In appendicitis due to *E. vermicularis*, various inflammatory patterns such as lymphoid hyperplasia and eosinophilic or neutrophilic infiltrate can be detected [34, 35]. However, in line with our results, reactive follicular hyperplasia without inflammation was the most prevalent pathological finding in the most reported cases [5, 29, 36]. Therefore, it seems that *E. vermicularis* is not directly involved in inflammatory appendicitis due to the lack of mucosal invasion. According to several studies, clinical symptoms of appendicitis appear to be caused by obstructing the appendix lumen by the accidental entrance of the adult worm rather than actual inflammation. However, regarding the debated role of *E. vermicularis* in appendicitis, the possible neglected role of this parasite should be considered [29, 36].

In the present study, *Enterobius* sp. was morphologically confirmed in the cross-sections mainly based on their cephalic alae located bilaterally along with the platymyarian and meromyarian types of their somatic muscles [35]. Molecular techniques have recently emerged as reliable identification tools to confirm microscopical parasitology. In the current study, we used nested PCR to amplify *E. vermicularis* *cox*1-gene from the old-archived appendix FFPE tissue. Archival FFPE tissues are the primary source of retrospective molecular studies; however, DNA extraction from them can be challenging because of the small size of the tissue and the insufficient quantities of DNA. Also, the cross-linking reaction of formaldehyde with DNA and protein could induce chemical modifications, DNA degradation, and fragmentation.

Furthermore, formalin can inhibit the proteinase K in the DNA extraction procedure and can interfere with PCR reaction by inhibiting DNA polymerase [37, 38]. Considering the old storage of blocks and the limitation of FFEP tissues, we used nested PCR with higher sensitivity. Of the 17 FFEP samples, 12 (70.6%) cases were amplified successfully by nested PCR (Fig 2); the earliest positive cases dated back to 1998. In 5 blocks, nested PCR was negative, possibly due to DNA fragmentation, long storage, or the small amount of tissue remaining in the blocks due to previous incisions. Owing to the high DNA fragmentation, successful amplification of DNA needs shorter amplification products, 250 to 300 bp [38]; however, a higher amplicon length was used to do appropriate phylogenetic analysis. In a survey by Rodriguez et al. [33], FFPE was used for molecular diagnosis of abdominal Angiostrongyliasis, in which 50% of confirmed pathological cases were positive by PCR. The promising results of nested PCR for detecting *E. vermicularis* in appendicitis tissue can be suggested this method as a complement to microscopy. Besides FFPE tissues, we used adult worms of *E. vermicularis* from 20 years ago, over-fixed in formalin; nonetheless, due to DNA fragmentation was failed to amplify.

Genetic diversity within the *cox*1 sequence (378 bp) of 12 isolates revealed six haplotypes. Seven sequences (five from Hamedan and two from Tehran) represent a single haplotype, proposing a common source of infection, whereas the other five sequences each represent a single haplotype (Table 2, Fig 3). Despite the lower number of cases from Tehran, the nucleotide diversity within the sequences from Tehran province was higher than from Hamadan, which could be a result of the fact that the higher diversity of patients from different geographical parts of Iran referred to the medical center in Tehran, the capital city of Iran. On the other hand, the least variation was seen within the isolates from Hamadan, where the population is more specific for that region, and less population mobility was seen.

The genetic distance among the samples varies from zero to 0.019. In this line, the intra-species variation among the Iranian population of *E. vermicularis* eggs was 0-1.2% in the Shiraz and Khorram Abad provinces of Iran [17]. In another study, the genetic difference among *E. vermicularis* isolates in European countries was reported as 1.2% to 2.1% [15]. Also, the highest divergence distance was observed between Mazandaran and Tehran (0.01161) samples and the lowest between Tehran and Hamedan (0.00525) samples, which could be related to the different geographical distances of the origin of isolates. This association between genetic diversity and geographical distance was also reported in other *E. vermicularis* population studies in Denmark, Greece, and Thailand [15, 18, 23]. In the *Enterobius* population from Denmark, the percentage of sequence difference (p-distance) increased gradually with the geographical distance by comparing Danish isolates with the German, Greek and Japanese isolates, 1.2%, 1.5%, and 4.2%, respectively. Thus, since *E. vermicularis* is mainly specific to humans, the genetic diversity between isolates of the same region or different countries varies depending on the geographical distance and population movement. [15]. Also, in the present study, the adult worm from Mazandaran exhibited the highest genetic distance (1.9%) comped to Tehran isolates; however, to clarify the potential differences between the *Enterobius* species that caused appendicitis and the other isolates that inhabit the lumen of the colon more samples should be studied.

In phylogenetic tree analysis, based on 87 *cox*1 sequences (333 bp), *E. vermicularis* was classified into three groups, A, B, and C, similar to the former studies [14, 39]. All 12 sequences from this study clustered in the type B category and were grouped in the same clade with previously reported sequences from Iran, Khorramabad province [17, 40]. On the other hand, the sequence from Tehran (MZ360958) was placed in a separate clade with sequences from Greece, Germany, and Iran (Fig 5). This diversity could be related to geographic regions, population movement, or high mutation rates in the *cox*1 gene [23].

Haplotypes network analysis of 87 *cox*1 sequences (333 bp) from different countries revealed 59 haplotypes, which were distributed based on *E. vermicularis*, geographical region, and population motility [15, 18]. In this network, five haplotypes were identified among the isolates compared with the GenBank sequences. Three haplotypes, including Hap57 (MZ361995), Hap58 (MZ361996), and Hap59 (MZ361999), are novel in type B and separate in different nodes, were each represented by a single sequence and belonged to the Hamden province. While two other haplotypes, Hap32 (MZ360958) and Hap37 (8 remaining sequences), were similar to the *E. vermicularis* population from Iran, Greece, and Germany (Fig 6).

The haplotype network, similar to the phylogenetic tree, demonstrated three different types. In agreement with the former studies, Type B is predominant in Europe and Asia and has been reported from humans in Iran, Greece, Germany, Thailand, Denmark, Sudan, the Czech Republic, and Poland, whereas it was isolated from chimpanzees in Japan [14-16, 23]. It was hypothesized that type B pinworms are probably specific to humans and possibly transmitted to captive chimpanzees from humans ([14, 39]. On the other hand, type A is less prevalent and was isolated from humans and chimpanzees in Japan and humans in Korea and Thailand [14, 41]. and Type C was just found in captive chimpanzees in Japan [14] (Fig 5, 6, Supplementary Table. S2).

Our results demonstrated that the average evolutionary divergence within the haplotypes of type B is higher than that of types A and C. Also, the highest divergence distance was observed between Type C and Type A (%6.35), and the lowest was between Type B and Type A (%3.46) haplotypes. Likewise, in a study by Nakano et al. 2006, a considerable distance of 1%, 4.1%, and 5.4% was detected for *E. vermicularis* types B, A, and C, respectively [14]. Furthermore, the p-distances between type A and C were reported in the range of 3.4%-5.8%, close to 6%, which is the maximum range seen within helminth species [13]. Since type C has just been reported from captive chimpanzees in Japan and has the highest diversity compared to other genotypes, analysis of different target genes is needed to clarify the evolutionary status of this cryptic genotype [14]. Also, given that *cox*1 sequences of *E. vermicularis* have been published from only a limited number of countries, more studies from different geographical regions from human and captive chimpanzees are needed to explore haplotypes circulating between populations and to shed light on the potential zoonotic cycle of *E. vermicularis*.

# Conclusion

In conclusion, *E. vermicularis* represents one of the controversial etiologies for acute appendicitis. Pathological findings demonstrated that non-inflammatory appendicitis is associated with *E. vermicularis*. Here, we report the molecular identification and phylogenetic analysis of *E. vermicularis* in Old-archived appendix FFPE tissue for the first time. The successful utilization of nested PCR on FFPE samples points to the need for further studies to explore the diagnostic performance of this technique in a prospective study based on routine pathology. Type B was identified in the samples of appendicitis using *cox*1 sequencing; however, more representatives from different geographical regions are needed to explore the possible genotype variations between the isolates found in appendicitis.

# Supporting information

**S1 Table. The result of the identity and coverage of the sequences obtained from the *cox*1 gene of this study with the isolates of the World Gene Bank. (DOC)**

**S1 Fig. Alignment of 378 bp of the nucleotide sequence of the *cox*1 gene fragment isolated from the paraffin blocks obtained from this study.** The presence of the asterisk indicates similarity, and its absence indicates the difference in the sequences in that position. **(DOC)**

**S2 Table. Sequences used in phylogeny tree, polymorphism, and haplotype network analysis according to haplotype cluster, host, and country. (DOC)**

# Funding

# This research has been supported by Tehran University of Medical Sciences & Health Services grant no. 1400-1-211-52257.

# Acknowledgments

We would like to acknowledge all colleagues of the pathology clinical Laboratory of Besat Hospital, Hamedan, Iran, and Children Medical Center Hospital, Tehran, Iran, for assistance in handling the samples. We thank Narges Anasori for helping with the histopathological processes.

# Authors' contributions

# Conceptualization: Gholamereza Mowlavi

# Data curation: Maryam Haghshenas, Alireza Latifi, Arash Dehghan, Bahram Nikmanesh,

# Formal analysis: Mona Koosha

# Funding acquisition: Gholamereza Mowlavi

# Investigation: Gholamereza Mowlavi

# Methodology: Mona Koosha, Maryam Haghshenas.

# Project administration: Mona Koosha

# Supervision: Gholamereza Mowlavi, Elham Kazemirad, Mona Koosha

# Writing – original draft: Mona Koosha, Alireza Latifi, Elham Kazemirad

# Writing – review & editing: Elham Kazemirad, Gholareza Mowlavi

# References

1.	Efared B, Atsame-Ebang G, Soumana BM, Tahiri L, Hammas N, El Fatemi H, et al. Acute suppurative appendicitis associated with Enterobius vermicularis: an incidental finding or a causative agent? A case report. BMC Research Notes. 2017;10(1):1-4. doi: <https://doi.org/10.1186/s13104-017-2822-3>. PMID: 28985767.

2.	Wendt S, Trawinski H, Schubert S, Rodloff AC, Mössner J, Lübbert C. The diagnosis and treatment of pinworm infection. Deutsches Ärzteblatt International. 2019;116(13):213. doi: <https://doi.org/10.3238/arztebl.2019.0213>. PMID: 31064642.

3.	Camacho M, Reinhard KJ. Confusing a pollen grain with a parasite egg: an appraisal of "Paleoparasitological evidence of pinworm (Enterobius vermicularis) infection in a female adolescent residing in ancient Tehran". The Korean Journal of Parasitology. 2019;57(6):621. doi: <https://doi.org/10.3347/kjp.2019.57.6.621>. PMID: 31914514.

4.	Guignard S, Arienti H, Freye L. Prevalance of enterobiosis in the children of Cordobe province. EJ Epidemiology. 2000;16(3):7. doi: <https://doi.org/10.1023/a:1007651714790>. PMID: 10870946.

5.	Mowlavi G, Massoud J, Mobedi I, Rezaian M, Mohammadi SS, Mostoufi N, et al. Enterobius vermicularis: a controversial cause of appendicitis. Iranian Journal of Public Health. 2004;33(3):27-31.

6.	Cateau E, Yacoub M, Tavilien C, Becq-Giraudon B, Rodier M-H. Enterobius vermicularis in the kidney: An unusual location. Journal of medical microbiology. 2010;59(7):860-1. doi: <https://doi.org/10.1099/jmm.0.019380-0>. PMID: 20378722.

7.	Akkuş S, Cıngıl DD. The effects of social-demographic characteristics and hygienic habits on the prevalence of Enterobius vermicularis in primary school children. Turkiye Parazitolojii Dergisi. 2005;29(1):39-42. PMID: 17167742.

8.	Selassie HG, Selassie HT, Ashebir D. Pattern and Outcome of Acute Appendicitis: Observational Prospective Study from a Teaching Hospital, Addis Ababa, Ethiopia. Open Access Emergency Medicine: OAEM. 2021;13:265. doi: <https://doi.org/10.2147/oaem.s315228>. PMID: 34188562.

9.	Addiss DG, Shaffer N, Fowler BS, Tauxe RV. The epidemiology of appendicitis and appendectomy in the United States. American journal of epidemiology. 1990;132(5):910-25. doi: <https://doi.org/10.1093/oxfordjournals.aje.a115734>. PMID: 2239906.

10.	Sodergren MH, Jethwa P, Wilkinson S, Kerwat R. Presenting features of Enterobius vermicularis in the vermiform appendix. Scandinavian journal of gastroenterology. 2009;44(4):457-61. doi: <https://doi.org/10.1080/00365520802624227>. PMID: 19085426.

11.	Blouin MS. Molecular prospecting for cryptic species of nematodes: mitochondrial DNA versus internal transcribed spacer. International journal for parasitology. 2002;32(5):527-31. doi: <https://doi.org/10.1016/s0020-7519(01)00357-5>. PMID: 11943225.

12.	Traversa D, Kuzmina T, Kharchenko VA, Iorio R, Klei TR, Otranto D. Haplotypic variability within the mitochondrial gene encoding for the cytochrome c oxidase 1 (cox1) of Cylicocyclus nassatus (Nematoda, Strongylida): evidence for an affiliation between parasitic populations and domestic and wild equid hosts. Veterinary parasitology. 2008;156(3-4):241-7. doi: <https://doi.org/10.1016/j.vetpar.2008.05.031>. PMID: 18619736.

13.	Blouin MS, Yowell CA, Courtney CH, Dame JB. Substitution bias, rapid saturation, and the use of mtDNA for nematode systematics. Molecular biology and evolution. 1998;15(12):1719-27. doi: <https://doi.org/10.1093/oxfordjournals.molbev.a025898>. PMID: 9866206.

14.	Nakano T, Okamoto M, Ikeda Y, Hasegawa H. Mitochondrial cytochrome c oxidase subunit 1 gene and nuclear rDNA regions of Enterobius vermicularis parasitic in captive chimpanzees with special reference to its relationship with pinworms in humans. Parasitology Research. 2006;100(1):51-7. doi: <https://doi.org/10.1007/s00436-006-0238-4>. PMID: 16788831.

15.	Ferrero MR, Röser D, Nielsen HV, Olsen A, Nejsum P. Genetic variation in mitochondrial DNA among Enterobius vermicularis in Denmark. Parasitology. 2013;140(1):109-14. doi: <https://doi.org/10.1017/s0031182012001308>. PMID: 22906211.

16.	Kubiak K, Dzika E. Enterobiasis epidemiology and molecular characterization of Enterobius vermicularis in healthy children in north-eastern Poland. Helminthologia. 2017;54(4):284-91.

17.	Tavan A, Mikaeili F, Mahmoudvand H, Sadjjadi SM, Bajelan S. Prevalence and genotype distribution of Enterobius vermicularis among kindergarteners in Shiraz and Khorramabad cities, Iran. Asian Pacific Journal of Tropical Medicine. 2020.

18.	Tomanakan K, Sanpool O, Chamavit P, Lulitanond V, Intapan P, Maleewong W. Genetic variation of Enterobius vermicularis among schoolchildren in Thailand. Journal of helminthology. 2020;94. doi: <https://doi.org/10.1017/s0022149x18000962>. PMID: 30369341.

19.	Ludyga N, Grünwald B, Azimzadeh O, Englert S, Höfler H, Tapio S, et al. Nucleic acids from long-term preserved FFPE tissues are suitable for downstream analyses. Virchows Archiv. 2012;460(2):131-40. doi: <https://doi.org/10.1007/s00428-011-1184-9>. PMID: 22270699.

20.	Dedhia P, Tarale S, Dhongde G, Khadapkar R, Das B. Evaluation of DNA extraction methods and real time PCR optimization on formalin-fixed paraffin-embedded tissues. Asian Pacific Journal of Cancer Prevention. 2007;8(1):55. PubMed PMID: 17477772.

21.	Garmaroudi AA, Khadem M, Hotkani MM, Mozaffari S, Kazemirad E. Downregulation of GJB2 and SLC26A4 genes induced by noise exposure is associated with cochlear damage. Molecular Biology Reports. 2022;49(8):7219-29. doi: <https://doi.org/10.1007/s11033-022-07291-7>. PMID: 35809183.

22.	Bessho Y, Ohama T, Osawa S. Planarian mitochondria I. Heterogeneity of cytochrome c oxidase subunit I gene sequences in the freshwater planarian, Dugesia japonica. Journal of molecular evolution. 1992;34(4):324-30. doi: <https://doi.org/10.1007/bf00160239>. PMID: 1314908.

23.	Piperaki E-T, Spanakos G, Patsantara G, Vassalou E, Vakalis N, Tsakris A. Characterization of Enterobius vermicularis in a human population, employing a molecular-based method from adhesive tape samples. Molecular and cellular probes. 2011;25(2-3):121-5. doi: <https://doi.org/10.1016/j.mcp.2011.03.005>. PMID: 21458560.

24.	Rozas J, Ferrer-Mata A, Sánchez-DelBarrio JC, Guirao-Rico S, Librado P, Ramos-Onsins SE, et al. DnaSP 6: DNA sequence polymorphism analysis of large data sets. Molecular biology and evolution. 2017;34(12):3299-302. doi: <https://doi.org/10.1093/molbev/msx248>. PMID: 29029172.

25.	Bandelt H-J, Forster P, Röhl A. Median-joining networks for inferring intraspecific phylogenies. Molecular biology and evolution. 1999;16(1):37-48. doi: <https://doi.org/10.1093/oxfordjournals.molbev.a026036>. PMID: 10331250.

26.	Kumar S, Stecher G, Li M, Knyaz C, Tamura K. MEGA X: molecular evolutionary genetics analysis across computing platforms. Molecular biology and evolution. 2018;35(6):1547. doi: <https://doi.org/10.1093/molbev/msy096>. PMID: 29722887.

27.	Kimura M. A simple method for estimating evolutionary rates of base substitutions through comparative studies of nucleotide sequences. Journal of molecular evolution. 1980;16(2):111-20.

28.	Humes D, Simpson J. Acute appendicitis. Bmj. 2006;333(7567):530-4. doi: <https://doi.org/10.1136/bmj.38940.664363.ae>. PMID: 16960208.

29.	Taghipour A, Olfatifar M, Javanmard E, Norouzi M, Mirjalali H, Zali MR. The neglected role of Enterobius vermicularis in appendicitis: A systematic review and meta-analysis. PloS one. 2020;15(4):e0232143. doi: <https://doi.org/10.1371/journal.pone.0232143>. PMID: 32324817.

30.	Ishiwata K, Shinohara A, Yagi K, Horii Y, Tsuchiya K, Nawa Y. Identification of tissue-embedded ascarid larvae by ribosomal DNA sequencing. Parasitology research. 2004;92(1):50-2. doi: <https://doi.org/10.1007/s00436-003-1010-7>. PMID: 14598166.

31.	Alarcón M, Pérez MC, Villarreal J, Araujo S, Goncalves L, González A, et al. Detection of Trypanosoma cruzi DNA in the placenta and fetuses of mice with Chagasic acute infection. Investigación Clínica. 2009;50(3):335-45. PMID: 19961056.

32.	Müller N, Zimmermann V, Forster U, Bienz M, Gottstein B, Welle M. PCR-based detection of canine Leishmania infections in formalin-fixed and paraffin-embedded skin biopsies: elaboration of a protocol for quality assessment of the diagnostic amplification reaction. Veterinary parasitology. 2003;114(3):223-9. doi: <https://doi.org/10.1016/s0304-4017(03)00139-0>. PMID: 12788257.

33.	Rodriguez R, da Silva ACA, Müller CA, Alves SL, Graeff-Teixeira C, Fornari F. PCR for the diagnosis of abdominal angiostrongyliasis in formalin-fixed paraffin-embedded human tissue. PloS one. 2014;9(4):e93658. doi: <https://doi.org/10.1371/journal.pone.0093658>. PMID: 24705328.

34.	Ariyarathenam A, Nachimuthu S, Tang T, Courtney E, Harris S, Harris A. Enterobius vermicularis infestation of the appendix and management at the time of laparoscopic appendicectomy: Case series and literature review. International Journal of Surgery. 2010;8(6):466-9. doi: <https://doi.org/10.1016/j.ijsu.2010.06.007>. PMID: 20637320.

35.	Lamps LW. Infectious causes of appendicitis. Infectious Disease Clinics. 2010;24(4):995-1018. doi: <https://doi.org/10.1016/j.idc.2010.07.012>. PMID: 20937462.

36.	Lala S, Upadhyay V. Enterobius vermicularis and its role in paediatric appendicitis: protection or predisposition? ANZ Journal of Surgery. 2016;86(9):717-9. doi: <https://doi.org/10.1111/ans.13464>. PMID: 26990375.

37.	Thavarajah R, Mudimbaimannar VK, Elizabeth J, Rao UK, Ranganathan K. Chemical and physical basics of routine formaldehyde fixation. Journal of oral and maxillofacial pathology: JOMFP. 2012;16(3):400. doi: <https://doi.org/10.4103/0973-029x.102496>. PMID: 23248474.

38.	Singh H, Narayan B, Urs AB, Polipalli SK, Kumar S. A novel approach for extracting DNA from formalin-fixed paraffin-embedded tissue using microwave. medical journal armed forces india. 2020;76(3):307-11. doi: <https://doi.org/10.1016/j.mjafi.2019.02.007>. PMID: 32773934.

39.	Hasegawa H, Sato H, Torii H. Redescription of Enterobius (Enterobius) macaci Yen, 1973 (Nematoda: Oxyuridae: Enterobiinae) based on material collected from wild Japanese macaque, Macaca fuscata (Primates: cercopithecidae). Journal of parasitology. 2012;98(1):152-9. doi: <https://doi.org/10.1645/ge-2867.1>. PMID: 21916620.

40.	Hagh VRH, Oskouei MM, Bazmani A, Miahipour A, Mirsamadi N. Genetic classification and differentiation of Enterobius vermicularis based on mitochondrial cytochrome c oxidase (cox1) in northwest of Iran. Journal of Pure and Applied Microbiology. 2014;8(5):3995-9.

41.	Kang S, Sultana T, Eom KS, Park YC, Soonthornpong N, Nadler SA, et al. The mitochondrial genome sequence of Enterobius vermicularis (Nematoda: Oxyurida)—an idiosyncratic gene order and phylogenetic information for chromadorean nematodes. Gene. 2009;429(1-2):87-97. doi: <https://doi.org/10.1016/j.gene.2008.09.011>. PMID: 18848867.
