# Supplementary material for: Detection of Enterobius vermicularis in archived formalin-fixed paraffin-embedded (FFPE) appendectomy blocks: It’s potential to compare genetic variations based on mitochondrial DNA (cox1) gene
Source: PLoS One. 2023 Feb 9;18(2):e0281622. doi: 10.1371/journal.pone.0281622 (PMC9910638; doi:10.1371/journal.pone.0281622)
Supplement: Attachment — Submitted filename: Response to Reviewers.docx [file pone.0281622.s006.docx]

**Dear Prof. Ebrahim Shokoohi**

**Editor of PLOS ONE	6- January -2023**

Referring to your email concerning our manuscript entitled: " **Detection of Enterobius vermicularis in archived formalin-fixed paraffin-embedded (FFPE) appendectomy blocks: It's potential to compare genetic variations based on mitochondrial DNA (cox1) gene,**" I would like to thank from your informative email and also from reviewers for their valuable and critical comments. Kindly inform you that all the following corrections and changes were done within the manuscript based on reviewers' suggestions and PLOS ONE's style. New and increased subjects have been highlighted in the manuscript. We hope the changes listed below meet your requirements.

Also, regarding the funder's role, we state, "The funders had no role in study design, data collection, and analysis, decision to publish, or preparation of the manuscript."

Overall, I deeply appreciate your consideration regarding this case and look forward to hearing from you.

Sincerely Yours,

Elham Kazemirad,

Corresponding Author, Assistant Professor of Medical Parasitology

Department of parasitology & Mycology, Tehran University of Medical Sciences,

Tel/Fax: +9821-88951392

[ekazemirad@yahoo.com](mailto:ekazemirad@yahoo.com) , [kazemirad@tuma.ac.ir](mailto:kazemirad@tuma.ac.ir)

**Editor comments:**

**1. Please ensure that your manuscript meets PLOS ONE's style requirements, including those for file naming.**

***Action/Comment***: We edited the manuscript based on PLOS ONE's style.

**2. We note that you have included the phrase "data not shown" in your manuscript. Unfortunately, this does not meet our data sharing requirements. PLOS does not permit references to inaccessible data. We require that authors provide all relevant data within the paper, Supporting Information files, or in an acceptable, public repository. Please add a citation to support this phrase or upload the data that corresponds with these findings to a stable repository (such as Figshare or Dryad) and provide and URLs, DOIs, or accession numbers that may be used to access these data. Or, if the data are not a core part of the research being presented in your study, we ask that you remove the phrase that refers to these data.**

***Action/Comment***: We omitted the phrase "data not shown." We wrote the results in the text and mentioned the divergence distance between the geographical regions in the text. Also, in phylogenetic analysis using different methods, including Maximum likelihood, neighbor-joining, and UPGMA analysis, a similar topology was generated among cox1 sequences. All the sentences were highlighted in gray color.

**3. Thank you for stating the following financial disclosure, "This research has been supported by Tehran University of Medical Sciences & health Services grant no. 1400-1-211-52257.”Please state what role the funders took in the study. If the funders had no role, please state: "The funders had no role in study design, data collection and analysis, decision to publish, or preparation of the manuscript."**

***Action/Comment***: We state that "The funders had no role in study design, data collection, and analysis, decision to publish, or preparation of the manuscript."

**4. Please upload a new copy of Figure 4 as the detail is not clear.**

***Action/Comment***: We upload the new high-qualified version of Figure 4.

**5. We note that Figure 6 in your submission contain [map/satellite] images which may be copyrighted. All PLOS content is published under the Creative Commons Attribution License (CC BY 4.0), which means that the manuscript, images, and Supporting Information files will be freely available online, and any third party is permitted to access, download, copy, distribute, and use these materials in any way, even commercially, with proper attribution. For these reasons, we cannot publish previously copyrighted maps or satellite images created using proprietary data, such as Google software (Google Maps, Street View, and Earth). For more information, see our copyright guidelines:** [**http://journals.plos.org/plosone/s/licenses-and-copyright**](http://journals.plos.org/plosone/s/licenses-and-copyright)**.**

***Action/Comment*:**

The map was displayed using the MARBLE package, and data comes from the OpenStreetMap database.

We used PopArt software to place sample locations for sequences on the map panel (http://popart.otago.ac.nz/index.shtml) (1). The map interface was implemented using the Marble library (http://marble.kde.org), which is distributed according to the GNU Lesser General Public License (LGPL), Version 2 (http://www.gnu.org/licenses/lgpl.html). Map data is copyright OpenStreetMap, and is used under the Open Database License (www.opendatacommons.org/licenses/odbl ).

The fig caption was edited and highlighted in yellow.

**Additional Editor Comments:**

**1-Why have you selected only cox1? Why not other genes included? Have you ever tested to be amplified by the PCR?**

***Action/Comment***: Thank you for raising this point.

Cytochrome c oxidase subunit 1 (*cox*1) gene is one of the most diverse mitochondrial genes used for the phylogenetic analysis of *E. vermicularis*. It can provide a more comprehensive representation of *E. vermicularis* evolution than other genes like ribosomal DNA (2, 3). In the previous studies in Japan and Thailand, partial sequences of ITS2 and Cytochrome c oxidase subunit 1 (*cox*1) genes were compared within *Enterobius vermicularis* isolates and demonstrated that ITS2 has a high similarity and low nucleotide variation to use in the phylogenetic tree. On the other hand, the mitochondrial cox1 gene revealed considerable genetic diversity in phylogenetic analysis. Consequently, the cox1 gene has been proposed as a discriminative marker for genetic diversity. Based on that, *E. vermicularis* is divided into three groups A, B, and C (4, 5). In most studies, the genetic diversity of *E. vermicularis* has been demonstrated according to the cox1 gene (6-8). In this line, we used this gene to demonstrate the potential genetic variation between the isolates extracted from archived FFPE samples and cox1 sequences deposited in the GenBank.

In this regard, we edited the introduction and emphasized more on the advantage of this gene for phylogenetic analysis. (Written in purple color)

**2-Figure 1, the scale should be in the same size and font.**

***Action/Comment***: We edited the Figure.

**2-Figure 2, is of low quality, and the authors mentioned 12 positive bands by PCR; why are only a few of them included?**

***Action/Comment***: As all PCR products were subjected to sequencing, we did PCR and gel electrophoresis again. The 1/100 dilution of DNA was used to eliminate the smears. We replaced the Figure, illustrated all the bands in two different agarose gels, and also edited the caption, written in red. Also, the not-cropped gel uploaded as S1_raw_images.

**3-Figure 4 is in low quality**

***Action/Comment***: We edited the Figure.

**4-In figure 5, the authors need to delete all ".1" from the accession numbers. Also, the worked sequences with accession numbers of the Iranian specimens should be given by the species name and in bold. You should also indicate the clade and discuss the clade with which your sequences are grouped with.**

***Action/Comment***: In Figure five, ".1" was deleted from the accession numbers, and the specimens used in the present study were **bolded**. We separated the groups in the Figure by different colors and highlighted the clades in which our isolates were placed. Also, we discussed other sequences that grouped with our samples. (Highlighted in turquoise color)

**5- In discussion, you need to discuss your sequences based on the histology, morphology, and similarity or dissimilarity with the relevant sequences.**

***Action/Comment***: Although histological and morphological characters are not discriminative among *E. vermicularis* isolates identified in FFPE appendectomies blocks, some limited variation was clarified within the isolates. In the present study, three new haplotypes were identified in type B, and also one sequence from Tehran (MZ360958) was placed in a separate clade with sequences from Greece, Germany, and Iran. According to the country's diverse geographical and climatological conditions, population movement, and tribe seasonal migration, much more extensive geographical sampling is required to illustrate a more phylogenetic informative pattern within E. vermicularis in the human population.

We added this statement in the discussion section and highlighted it in green.

**6-The references need to be adjusted in font and style with PLoS One.**

***Action/Comment***: We edited the manuscript based on PLOS ONE style. Also, the whole manuscript was revised grammatically, with all the changes written in blue.

**7- the rest of the comments are included in the attached file.
*Action/Comment***: All the remaining comments were replied to in the manuscript.

[Note: HTML markup is below. Please do not edit.]

**Reviewers' comments:**

Reviewer's Responses to Questions

**Comments to the Author**

1. Is the manuscript technically sound, and do the data support the conclusions?

The manuscript must describe a technically sound piece of scientific research with data that supports the conclusions. Experiments must have been conducted rigorously, with appropriate controls, replication, and sample sizes. The conclusions must be drawn appropriately based on the data presented.

Reviewer #1: Yes

2. Has the statistical analysis been performed appropriately and rigorously?

Reviewer #1: Yes

3. Have the authors made all data underlying the findings in their manuscript fully available?

The [PLOS Data policy](http://www.plosone.org/static/policies.action#sharing) requires authors to make all data underlying the findings described in their manuscript fully available without restriction, with rare exception (please refer to the Data Availability Statement in the manuscript PDF file). The data should be provided as part of the manuscript or its supporting information, or deposited to a public repository. For example, in addition to summary statistics, the data points behind means, medians and variance measures should be available. If there are restrictions on publicly sharing data—e.g. participant privacy or use of data from a third party—those must be specified.

Reviewer #1: Yes

4. Is the manuscript presented in an intelligible fashion and written in standard English?

PLOS ONE does not copyedit accepted manuscripts, so the language in submitted articles must be clear, correct, and unambiguous. Any typographical or grammatical errors should be corrected at revision, so please note any specific errors here.

Reviewer #1: No

5. Review Comments to the Author

Please use the space provided to explain your answers to the questions above. You may also include additional comments for the Author, including concerns about dual publication, research ethics, or publication ethics. (Please upload your review as an attachment if it exceeds 20,000 characters)

Reviewer #1: The manuscript including data of mtDNA of preserved specimens from Iran. The manuscript includes such interesting data, but it needs to be improved.

**1-why authors selected coxi? Why other genes not been selected?**

***Action/Comment***: Thanks for the precise question.

Cytochrome c oxidase subunit 1 (*cox*1) gene is one of the most diverse mitochondrial genes used for the phylogenetic analysis of *E. vermicularis*. It can provide a more comprehensive representation of *E. vermicularis* evolution than other genes like ribosomal DNA (2, 3). In the previous studies in Japan and Thailand, partial sequences of ITS2 and Cytochrome c oxidase subunit 1 (*cox*1) genes were compared within *Enterobius vermicularis* isolates and demonstrated that ITS2 has a high similarity and low nucleotide variation to use in the phylogenetic tree. On the other hand, the mitochondrial cox1 gene revealed considerable genetic diversity in phylogenetic analysis. Consequently, the cox1 gene has been proposed as a discriminative marker for genetic diversity. Based on that, *E. vermicularis* is divided into three groups A, B, and C (4, 5). In most studies, the genetic diversity of *E. vermicularis* has been demonstrated according to the cox1 gene (6-8). In this line, we used this gene to demonstrate the potential genetic variation between the isolates extracted from archived FFPE samples and cox1 sequences deposited in the GenBank.

In this regard, we edited the introduction and emphasized more on the advantage of this gene for phylogenetic analysis. (Written in purple color)

**2-detail method of extraction from formalin fixed specimens not explained well.**

***Action/Comment***: We explained the detail of DNA extraction from FFPE tissue, highlighted in pink.

**3-in line 304, the Author mentioned data not shown. Why? it should be presented or provided as supplementary file.**

***Action/Comment***: We omitted the phrase "data not shown." We wrote the results in the text and mentioned the divergence distance between the geographical regions in the text. Also, in phylogenetic analysis using different methods, including Maximum likelihood, neighbor-joining, and UPGMA analysis, a similar topology was generated among cox1 sequences. All the sentences were highlighted in gray color.

**4-line 271 to 272 need to be revised, it is unclear.**

***Action/Comment***: It was revised and written in orange color.

**5-English style needs to be improved.**

***Action/Comment***: It was edited, and all the new editions and changes were written in blue.

**6-The Figure are not in high quality and should be improved.**

***Action/Comment***: All the figures were improved and replaced.

6. PLOS authors have the option to publish the peer review history of their article ([what does this mean?](https://journals.plos.org/plosone/s/editorial-and-peer-review-process#loc-peer-review-history)). If published, this will include your full peer review and any attached files.


If you choose "no", your identity will remain anonymous but your review may still be made public.

**Do you want your identity to be public for this peer review?** For information about this choice, including consent withdrawal, please see our [Privacy Policy](https://www.plos.org/privacy-policy).

Reviewer #1: No

[NOTE: If reviewer comments were submitted as an attachment file, they will be attached to this email and accessible via the submission site. Please log into your account, locate the manuscript record, and check for the action link "View Attachments". If this link does not appear, there are no attachment files.]

While revising your submission, please upload your figure files to the Preflight Analysis and Conversion Engine (PACE) digital diagnostic tool, <https://pacev2.apexcovantage.com/>. PACE helps ensure that figures meet PLOS requirements. To use PACE, you must first register as a user. Registration is free. Then, login and navigate to the UPLOAD tab, where you will find detailed instructions on how to use the tool. If you encounter any issues or have any questions when using PACE, please email PLOS at [figures@plos.org](mailto:figures@plos.org). Please note that Supporting Information files do not need this step.

**References**

1. Leigh JW, Bryant D. POPART: full-feature software for haplotype network construction. Methods Ecol Evol. 2015; 6:1110-6.
2. Blouin MS. Molecular prospecting for cryptic species of nematodes: mitochondrial DNA versus internal transcribed spacer. Int J Parasitol. 2002; 32:527-31. <https://doi.org/10.1016/s0020-7519(01)00357-5> PMID: [11943225](https://pubmed.ncbi.nlm.nih.gov/11943225/).
3. Kang S, Sultana T, Eom KS, Park YC, Soonthornpong N, Nadler SA, Park JK. The mitochondrial genome sequence of Enterobius vermicularis (Nematoda: Oxyurida)--an idiosyncratic gene order and phylogenetic information for chromadorean nematodes. Gene. 2009; 429:87-97. <https://doi.org/10.1016/j.gene.2008.09.011> PMID: [18848867](https://pubmed.ncbi.nlm.nih.gov/18848867/).
4. Nakano T, Okamoto M, Ikeda Y, Hasegawa H. Mitochondrial cytochrome c oxidase subunit 1 gene and nuclear rDNA regions of Enterobius vermicularis parasitic in captive chimpanzees with special reference to its relationship with pinworms in humans. Parasitol Res. 2006; 100:51-7. <https://doi.org/10.1007/s00436-006-0238-4> PMID: [16788831](https://pubmed.ncbi.nlm.nih.gov/16788831/).
5. Tomanakan K, Sanpool O, Chamavit P, Lulitanond V, Intapan PM, Maleewong W. Genetic variation of Enterobius vermicularis among schoolchildren in Thailand. J Helminthol. 2018; 94:e7. <https://doi.org/10.1017/s0022149x18000962> PMID: [30369341](https://pubmed.ncbi.nlm.nih.gov/30369341/).
6. Ferrero MR, Röser D, Nielsen HV, Olsen A, Nejsum P. Genetic variation in mitochondrial DNA among Enterobius vermicularis in Denmark. Parasitology. 2013; 140:109-14. <https://doi.org/10.1017/s0031182012001308> PMID: [22906211](https://pubmed.ncbi.nlm.nih.gov/22906211/).
7. Kubiak K, Dzika E. Enterobiasis epidemiology and molecular characterization of Enterobius vermicularis in healthy children in north-eastern Poland. Helminthologia. 2017; 54:284-91.
8. Hagh VR, Oskouei MM, Bazmani A, Miahipour A, Mirsamadi N. Genetic classification and differentiation of Enterobius vermicularis based on mitochondrial cytochrome c oxidase (cox1) in northwest of Iran. J Pure Appl Microbiol. 2014; 8:3995-9.
